# Altered Gene Expression in Prefrontal Cortex of a Fabry Disease Mouse Model

**DOI:** 10.3389/fnmol.2018.00201

**Published:** 2018-06-25

**Authors:** Kai K. Kummer, Theodora Kalpachidou, Miodrag Mitrić, Michiel Langeslag, Michaela Kress

**Affiliations:** Division of Physiology, Department of Physiology and Medical Physics Medical, University of Innsbruck, Innsbruck, Austria

**Keywords:** Fabry disease, alpha Galactosidase A, lysosomal storage disorder, prefrontal cortex, neuropathic pain, cognitive deficits

## Abstract

Fabry disease is an X-chromosome linked hereditary disease that is caused by loss of function mutations in the α-galactosidase A (α-Gal A) gene, resulting in defective glycolipid degradation and subsequent accumulation of globotriaosylceramide (Gb3) in different tissues, including vascular endothelial cells and neurons in the peripheral and central nervous system. We recently reported a differential gene expression profile of α-Gal A^(−/0)^ mouse dorsal root ganglia, an established animal model of Fabry disease, thereby providing new gene targets that might underlie the neuropathic pain related symptoms. To investigate the cognitive symptoms experienced by Fabry patients, we performed one-color based hybridization microarray expression profiling of prefrontal cortex samples from adult α-Gal A^(−/0)^ mice and age-matched wildtype controls, followed by protein-protein interaction and pathway analyses for the differentially regulated mRNAs. We found that from a total of 381 differentially expressed genes, 135 genes were significantly upregulated, whereas 246 genes were significantly downregulated between α-Gal A^(−/0)^ mice and wildtype controls. Enrichment analysis for downregulated genes revealed mainly immune related pathways, including immune/defense responses, regulation of cytokine production, as well as signaling and transport regulation pathways. Further analysis of the regulated genes revealed a large number of genes involved in neurodegeneration. The current analysis for the first time presents a differential gene expression profile of central nervous system tissue from α-Gal A^(−/0)^ mice, thereby providing novel knowledge on the deregulation and a possible contribution of gene expression to Fabry disease related brain pathologies.

## Introduction

Fabry disease (FD) is an X-chromosome linked hereditary disease that belongs to the group of lysosomal storage disorders. It is caused by loss of function mutations in the lysosomal α-galactosidase A (α-Gal A) gene that result in defective glycolipid degradation, and subsequent accumulation of globotriaosylceramide (Gb3) in different tissues, including vascular endothelial cells and neurons (Desnick et al., 2001; Gal et al., 2006; Saito et al., 2011; Bangari et al., 2015). Incidence rates for FD span from 1:37′000 for the classical phenotype to 1:3′100 for a late-onset disease variant, with males being more affected than females (Spada et al., 2006; Mechtler et al., 2012). Nevertheless, also heterozygous females show variable expression of α-Gal A caused by random X-inactivation, which leads to major organ involvement (Wilcox et al., 2008). One of the earliest symptoms of FD is small-fiber neuropathy, which leads to pain attacks already in childhood and is associated with accumulation of Gb3 in sensory neurons of dorsal root ganglia (DRG) (Germain, 2010; Bangari et al., 2015). In addition to neuronal accumulation in the peripheral nervous system, Gb3 deposits are also found in neurons and other cell types of the central nervous system (Khanna et al., 2010; Tuttolomondo et al., 2013a,b). Often, those glycolipid deposits lead to alterations in cerebral vessels and the formation of microstructural damage (i.e., microbleeds) in different brain regions (Tagliavini et al., 1982; Reisin et al., 2011; Paavilainen et al., 2013; Kono et al., 2016). In line with these pathologies, different forms of cognitive deficits have been reported in FD patients, spanning from higher prevalence of depression and anxiety to impairment of information processing and attention (Schermuly et al., 2011; Bolsover et al., 2014; Sigmundsdottir et al., 2014), as well as impaired long-term verbal memory (Cocozza et al., 2018). These are reflected to some extent in a transgenic FD mouse model which shows increased anxiety-like behavior (Hofmann et al., 2017).

In a recent microarray profiling study of DRGs, we provide the first report on changed gene expression in neuronal tissue from a mouse line with a null mutation of α-Gal A modeling FD (Kummer et al., 2017). Besides the identification of numerous regulated genes, enriched pathways are implicated in general neuronal dysfunction, for example G-protein coupled receptor activity and neuropeptide signaling that might affect excitability and neuronal activation in FD (Kummer et al., 2017). The above mentioned cognitive deficits seen in FD patients and mice may be related to such changes in neuronal excitability or compromised receptor activity in higher brain regions, most probably in the prefrontal cortex (PFC), as the major deficits reported from FD patients refer to attention and executive functioning.

We therefore set out to explore neuronal gene expression changes associated with the loss of function of α-Gal A in a particularly important brain area and link it to the reported cognitive deficits, by performing mRNA microarray expression profiling of PFC tissue samples from adult male α-Gal A^(−/0)^ mice, followed by qPCR validation and in depth bioinformatics analyses of protein-protein interactions and pathways.

## Methods

### Animals

Male α-galactosidase A^(−/0)^ (α-Gal A^(−/0)^; background C57BL/6; kindly provided by Dr. A. Kulkarni, National Institute of Health, NIDCR, Bethesda, USA) (Ohshima et al., 1997) and wildtype C57BL/6J mice (age 20-24 weeks) were inbred and housed under specific pathogen-free (SPF) conditions. For microarray expression profiling mice from the separate inbred colonies were used, whereas for RT-qPCR validation, α-Gal A^(−/0)^ mice backcrossed with wildtype C57BL/6J mice and wildtype C57BL/6J mice were used to control for inbred colony effects. Animals were maintained at constant room temperature of 24°C on a 12h light/dark cycle with lights on from 07:00 to 19:00 and had *ad libitum* access to autoclaved pelleted food and water. All animals were treated in accordance with the Ethics Guidelines of Animal Care (Medical University of Innsbruck), as well as the European Communities Council Directive of 22 September 2010 on the protection of animals used for scientific purposes (2010/63/EU), and approved by the Austrian National Animal Experiment Ethics Committee of the Austrian Bundesministerium für Wissenschaft und Forschung (permit number BMWF-66.011/0054-WF/V/3b/2015).

### Tissue collection

For microarray expression profiling four adult mice per group, and for RT-qPCR validation six adult mice per group were deeply anesthetized with isoflurane and euthanized by decapitation. Brains were removed, prefrontal cortices dissected and flash-frozen in liquid nitrogen. Samples were stored at −80°C until further processing.

### Microarray expression profiling

Genome-wide expression profiling was carried out by IMGM Laboratories (Munich, Germany) using Agilent SurePrint G3 Mouse GE 8x60K Microarrays in combination with a one-color based hybridization protocol. Microarray signals were detected using the Agilent DNA Microarray Scanner.

Total RNA including small RNAs was isolated using the miRNeasy Mini Kit (Qiagen) according to the manufacturer's instructions and eluted in 40 μl RNase-free water. RNA concentration and purity was determined on a NanoDrop ND-1000 spectral photometer (Peqlab). Samples were analyzed using the RNA 6000 Nano LabChip Kit (Agilent Technologies) on a 2100 Bioanalyzer (Agilent Technologies). For mRNA analysis, total RNA samples were spiked with *in vitro* synthesized polyadenylated transcripts (One-Color RNA Spike-In Mix, Agilent Technologies), reverse transcribed into cDNA and then converted into Cyanine-3 labeled complementary RNA (Low Input Quick-Amp Labeling Kit One-Color, Agilent Technologies) according to the manufacturer's instructions. cRNA concentration, RNA absorbance ratio and Cyanine-3 dye concentration were recorded using a NanoDrop ND-1000 UV-VIS spectral photometer, and quality of labeled cRNA was analyzed using the RNA 6000 Nano LabChip Kit (Agilent Technologies) on a 2100 Bioanalyzer (Agilent Technologies). Following cRNA clean-up and quantification, Cyanine-3-labeled cRNA samples were fragmented and prepared for one-color-based hybridization (Gene Expression Hybridization Kit, Agilent Technologies) and hybridized at 65^°^C for 17 h on Agilent SurePrint G3 Mouse GE 8x60K Microarrays. After hybridization, microarrays were washed with increasing stringency using Triton X-102 supplemented Gene Expression Wash Buffers (Agilent Technologies) followed by drying with acetonitrile (Sigma). Fluorescence signals were detected on an Agilent DNA Microarray Scanner and extracted using feature extraction software (Agilent Technologies). The data discussed in this publication have been deposited in NCBI's Gene Expression Omnibus (Edgar et al., 2002) and are accessible through GEO Series accession number GSE110645 (https://www.ncbi.nlm.nih.gov/geo/query/acc.cgi?acc=GSE110645).

### RT-qPCR validation of regulated genes

Reverse transcription quantitative polymerase chain reaction (RT-qPCR) validation of regulated genes was performed using TaqMan Gene Expression Assays (Thermo Fisher Scientific) in an Applied Biosystems 7500 Fast Real-Time PCR System (Thermo Fisher Scientific).

Total RNA was extracted using peqGOLD TriFast reagent (Peqlab) according to the manufacturer's instructions. The quality and quantity of RNA was evaluated using NanoDrop 2000 (Thermo Scientific). Reverse transcription of mRNA was performed as previously described (Kummer et al., 2017). Genes of interest were analyzed by RT-qPCR using the following TaqMan Gene Expression Assays (Thermo Fisher Scientific): Mm00499982_m1 (Cdhr1), Mm01207095_g1 (Agfg2), Mm01297785_m1 (Cpne5), Mm00839582_m1 (Dynlt1a/1b/1c/1f), Mm00723335_m1 (Fam83a), Mm00619552_m1 (Fn3krp), Mm00446358_m1 (Fxyd2), Mm03646971_gH (Gm1987), Mm02391771_g1 (Hdac1), Mm01215650_m1 (Kcnj6), Mm01188211_m1 (S100pbp), Mm00555295_m1 (Sc5d), Mm00503605_m1 (Tmem25), Mm00836474_m1 (Zfp932), Mm00446968_m1 (Hprt), Mm01352363_m1 (Sdha), and Mm00441941_m1 (Tfrc). Experimental procedures were performed according to the TaqMan Gene Expression Assays protocol. The reactions were loaded on MicroAmp Fast Optical 96-well reaction plates (Thermo Fisher Scientific) and placed in the Applied Biosystems 7500 Fast Real-Time PCR System (Thermo Fisher Scientific). The PCR cycle protocol used was: 10 min at 95^°^C, 40 two-step cycles of 15 s at 95^°^C and 1 min at 60^°^C. Each sample was run in duplicates alongside non-template controls. Threshold was set manually at 0.1 and threshold cycle (C_T_) was used as a measure of initial RNA input. Relative fold changes in gene expression were calculated using the 2^−ΔΔ*CT*^ method. All fold changes were expressed relative to the respective expression in wildtype mice and analyzed using Welch's *t*-test. Three genes (i.e., Hprt, Sdha, and Tfrc) were used as reference genes. All three reference genes were found to be stably expressed in both groups of animals, as indicated by geNorm, Normfinder, and Bestkeeper software packages.

### Bioinformatics analyses

GeneSpring GX 13.0 analysis software (Agilent Technologies) was used to normalize and analyze the microarray raw data. Data were normalized using non-parametric quantile normalization. Groups were compared using Welch's approximate *t*-test (unpaired unequal variances) and *p*-values corrected for multiple testing using the algorithm of Benjamini and Hochberg (Benjamini and Hochberg, 1995), controlling for false discovery rate (FDR). Differential expression between the two groups was determined by calculating fold changes of the averaged normalized expression values. Significantly regulated mRNAs were identified by applying filters on fold changes (absolute fold change ≥ 1.2 or ≥ 2) and p-values (*p* ≤ 0.01). Gene expression data were further processed by *R statistics* statistical software package (R Development Core Team, 2008) and Volcano plots prepared using R statistics *ggplot* library. Only genes with uncompromised hybridization values in all individual samples were used for the current analysis.

### Protein-protein interaction analysis

Protein-protein interactions were investigated for the significantly regulated mRNAs using the STRING Database v. 10.5 (http://www.string-db.org) (Szklarczyk et al., 2017), which includes direct and indirect protein associations collected from different databases. Interaction networks were prepared using medium confidence scores (0.40) and clustered using MCL clustering algorithm (inflation parameter: 3). Disconnected nodes were hidden from the network.

### Functional enrichment and pathway analyses

Functional enrichment and pathway analyses were also performed using the STRING Database v. 10.5 (http://www.string-db.org). Classification systems tested were Gene Ontology and KEGG functional annotation spaces, employing Fisher's exact test followed by FDR correction for multiple testing. Only enriched pathways with FDR corrected *p*-values < 0.05 are reported.

## Results

### mRNA expression profile of fabry mouse PFC

**Figure 1:**
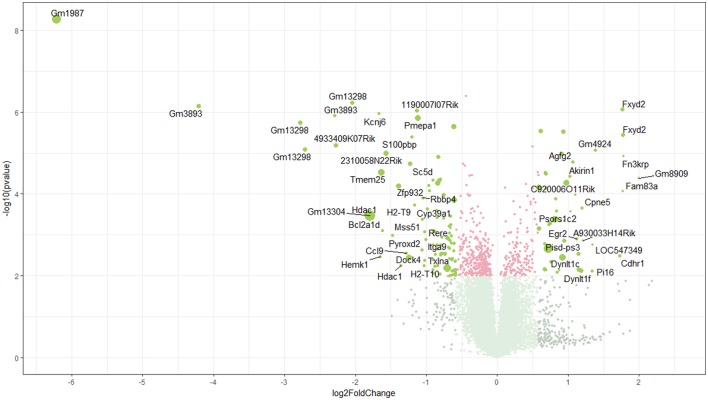
Volcano plot of microarray data. Green color, *p* ≤ 0.01, fold change ≥ 1.2; labels, *p* ≤ 0.01, fold change ≥ 2.0; dot size represents relative expression values of wildtype mice.

**Figure 2:**
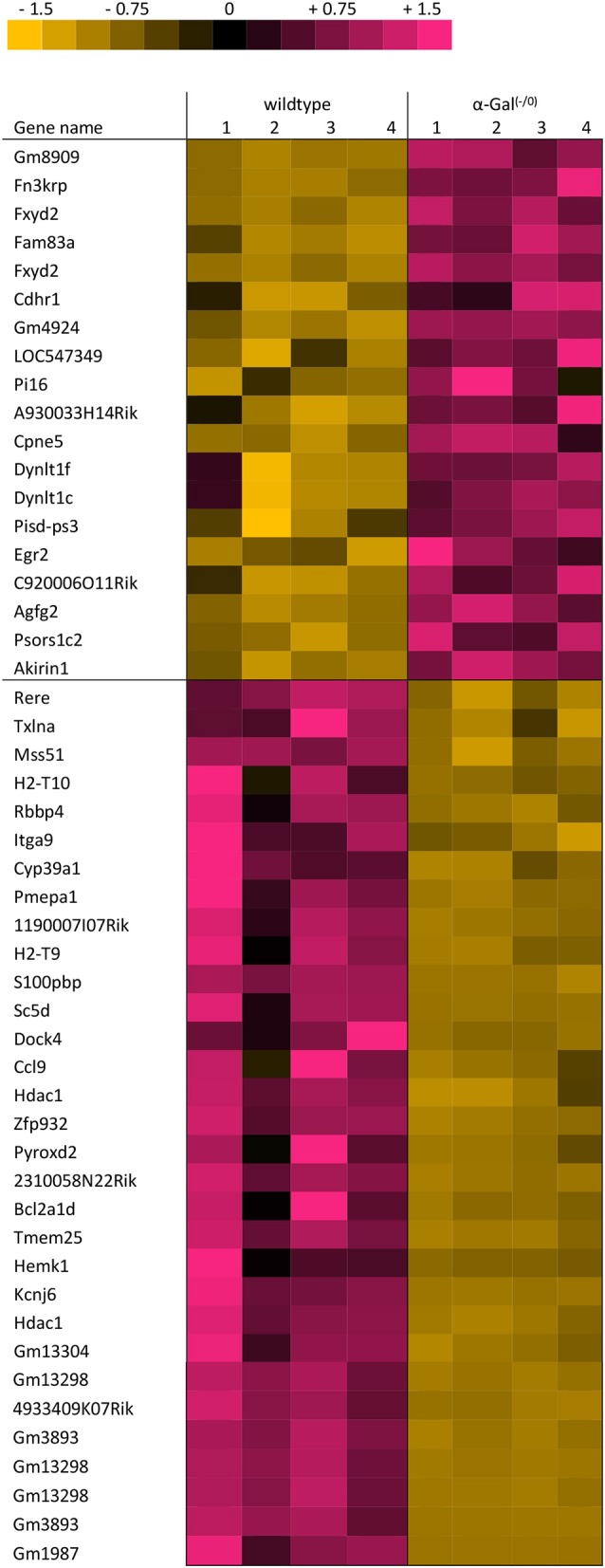
Heatmap of significantly regulated genes. Cut-off values, *p* ≤ 0.01, fold change ≥ 2.

Using microarray expression profiling we found that in total 381 genes from the overall 21′393 detected mRNAs were significantly different between PFC samples from wildtype and α-Gal A^(−/0)^ mice (criteria *p* ≤ 0.01, absolute fold change ≥ 1.2) (Figure 1). Of those, 135 genes were significantly upregulated and 246 genes were significantly downregulated as compared to wildtype controls. More stringent filtering (criteria *p* ≤ 0.01, absolute fold change ≥ 2) of the significantly regulated genes revealed an assessable number of 50 genes in total (Figure 2). Using these criteria 19 genes were significantly upregulated, of which 11 showed FDR corrected *p* ≤ 0.1 (Table 1). Furthermore, 31 genes were significantly downregulated, of which 17 showed FDR corrected *p* ≤ 0.1 (Table 2). Protein-protein interaction (PPI) analysis (STRING Database) did not reveal clusters of interacting proteins or enriched pathways when applying the stringent filtering criteria, due to the low number of input genes. Therefore, less stringent filtering criteria (criteria *p* ≤ 0.01, absolute fold change ≥ 1.2) were used for PPI and enrichment analyses.

**Table 1:** Raw expression values, fold changes, and statistical analysis for significantly *upregulated* genes.

**NCBI refSeq ID**	**Gene symbol**	**Gene name**	**Chromosome and location**	**Expression α-Gal^(–/0)^**	**Expression wildtype**	**Fold change**	***p*-value**	**FDR**
NM_001081032	Gm8909	Predicted gene 8909	Chr17: 36302361–36302302	225	63	4.0	< 0.0001	0.0288
NM_181420	Fn3krp	Fructosamine 3 kinase related protein	Chr11: 121292014–121292073	252	82	3.4	< 0.0001	0.0141
NM_052823	Fxyd2	FXYD domain-containing ion transport regulator 2	Chr9: 45218302–45218361	5394	1830	3.4	< 0.0001	0.0077
BC030396	Fam83a	Family with sequence similarity 83, member A	Chr15: 57827097–57827156	82	27	3.4	< 0.0001	0.0439
NM_052823	Fxyd2	FXYD domain-containing ion transport regulator 2	Chr9: 45218281–45218340	5702	1948	3.4	< 0.0001	0.0050
NM_130878	Cdhr1	Cadherin-related family member 1	Chr14: 37891446–37891387	2986	1034	3.3	0.0033	0.2910
DQ459435	Gm4924	Predicted gene 4924	Chr10: 81863532–81863591	2454	1076	2.6	< 0.0001	0.0135
NM_001025208	LOC547349	MHC class I family member	–	103	45	2.5	0.0017	0.2337
NM_023734	Pi16	Peptidase inhibitor 16	Chr17: 29465784–29465843	367	163	2.5	0.0076	0.3873
XR_105403	A930033H14Rik	RIKEN cDNA A930033H14 gene	Chr10: 68672629–68672570	182	87	2.3	0.0014	0.2149
NM_153166	Cpne5	Copine V	Chr17: 29293557–29293498	591	291	2.3	< 0.0001	0.0827
NM_001199948	Dynlt1f	Dynein light chain Tctex-type 1F	Chr17: 6606983–6607042	5258	2692	2.3	0.0076	0.3873
NM_001166630	Dynlt1c	Dynein light chain Tctex-type 1C	Chr17: 6812527–6812586	7657	4016	2.2	0.0072	0.3839
NR_003518	Pisd-ps3	Phosphatidylserine decarboxylase, pseudogene 3	Chr11: 003030755–003030814	3473	1816	2.2	0.0028	0.2815
NM_010118	Egr2	Early growth response 2	Chr10: 67004798–67004857	546	281	2.2	0.0012	0.2052
NR_040401	C920006O11Rik	RIKEN cDNA C920006O11 gene	Chr9: 78026086–78026145	63	33	2.1	< 0.0001	0.0510
NM_145566	Agfg2	ArfGAP with FG repeats 2	Chr5: 138104107–138104048	1041	562	2.1	< 0.0001	0.0176
NM_020576	Psors1c2	Psoriasis susceptibility 1 candidate 2 (human)	Chr17: 35671531–35671590	121	66	2.1	< 0.0001	0.0906
NM_023423	Akirin1	Akirin 1	Chr4: 123420761–123420702	1688	945	2.0	< 0.0001	0.0283

*Genes with FDR-corrected p-values ≥ 0.1 are grayed out*.

**Table 2:** Raw expression values, fold changes, and statistical analysis for significantly *downregulated* genes.

**NCBI refSeq ID**	**Gene symbol**	**Gene name**	**Chromosome and location**	**Expression α-Gal^(–/0)^**	**Expression wildtype**	**Fold change**	***p*-value**	**FDR**
NM_001193667	Gm1987	Predicted gene 1987	chr4: 42232117-−42232176	263	22237	−74.4	< 0.0001	0.0002
NR_033506	Gm3893	Predicted gene 3893	chrUn_random: 553693–553634	173	3633	−18.4	< 0.0001	0.0050
NM_001085530	Gm13298	Predicted gene 13298	chr4: 41842107–41842166	455	3557	−6.8	< 0.0001	0.0061
NM_001085530	Gm13298	Predicted gene 13298	chr4: 41841838-−41841897	491	3682	−6.5	< 0.0001	0.0135
NR_033506	Gm3893	Predicted gene 3893	chrUn_random: 551587-−551528	182	1003	−4.9	< 0.0001	0.0050
NR_033123	4933409K07Rik	RIKEN cDNA 4933409K07 gene	chr4: 42472710–42472769	566	3139	−4.8	< 0.0001	0.0127
NM_001085530	Gm13298	Predicted gene 13298	chr4: 41841716–41841775	324	1512	−4.1	< 0.0001	0.0050
NM_001193666	Gm13304	Predicted gene 13304	chr4: 41775274–41775333	9111	36638	−3.5	0.0003	0.1037
NM_008228	Hdac1	Histone deacetylase 1	chr4: 129193586–129193527	551	2160	−3.4	0.0003	0.1053
NM_001025585	Kcnj6	Potassium inwardly-rectifying channel, subfamily J, member 6	chr16: 95054032–95053973	91	322	−3.2	< 0.0001	0.0050
NM_133984	Hemk1	HemK methyltransferase family member 1	chr9: 107233482–107233423	70	247	−3.1	0.0035	0.2946
NM_027865	Tmem25	Transmembrane protein 25	chr9: 44602002–44601943	2483	9033	−3.1	< 0.0001	0.0257
NM_007536	Bcl2a1d	B cell leukemia/lymphoma 2 related protein A1d	chr9: 88618224–88618165	99	340	−3.1	0.0008	0.1665
AK009987	2310058N22Rik	RIKEN cDNA 2310058N22 gene	chr12: 117619193–117619252	1674	5754	−3.0	< 0.0001	0.0135
NM_029011	Pyroxd2	Pyridine nucleotide-disulphide oxidoreductase domain 2	chr19: 42801304–42801245	70	218	−2.8	0.0010	0.1859
NM_145563	Zfp932	Zinc finger protein 932	chr5: 110439205–110439264	1479	4497	−2.6	0.0001	0.0361
NM_008228	Hdac1	Histone deacetylase 1	chr4: 129193775–129193716	154	444	−2.6	0.0057	0.3473
NM_011338	Ccl9	Chemokine (C-C motif) ligand 9	chr11: 83388282–83388223	78	212	−2.4	0.0028	0.2797
NM_172803	Dock4	Dedicator of cytokinesis 4	chr12: 41572977–41573036	2655	7324	−2.4	0.0037	0.2999
NM_172769	Sc5d	Sterol-C5-desaturase (fungal ERG3, delta-5-desaturase) homolog (S. cerevisae)	chr9: 42062916–42062857	1191	3202	−2.3	< 0.0001	0.0186
AK139097	S100pbp	S100P binding protein	chr4: 128854488–128854429	259	670	−2,3	< 0.0001	0,0083
NM_010399	H2-T9	Histocompatibility 2, T region locus 9	chr17: 36177230–36177171	90	226	−2.2	0.0002	0.0714
NM_001135567	1190007I07Rik	RIKEN cDNA 1190007I07 gene	chr10: 82082933–82082874	854	2138	−2.2	< 0.0001	0.0050
NM_022995	Pmepa1	Prostate transmembrane protein, androgen induced 1	chr2: 173050025–173049966	2568	6494	−2.2	< 0.0001	0.0051
NM_018887	Cyp39a1	Cytochrome P450, family 39, subfamily a, polypeptide 1	chr17: 43887735–43887794	225	527	−2.1	0.0004	0.1160
NM_133721	Itga9	Integrin alpha 9	chr9: 118807900–118807959	273	637	−2.1	0.0024	0.2622
NM_009030	Rbbp4	Retinoblastoma binding protein 4	chr4: 128984682–128984623	149	345	−2.1	0.0001	0.0567
NM_010395	H2-T10	Histocompatibility 2, T region locus 10	chr17: 36255977–36255918	87	200	−2.0	0.0056	0.3473
NM_029104	Mss51	MSS51 mitochondrial translational activator	chr14: 21302782–21302415	104	238	−2.0	0.0008	0.1697
NM_001005506	Txlna	Taxilin alpha	chr4: 129316603–129316544	80	182	−2.0	0.0041	0.3101
NM_001085492	Rere	Arginine glutamic acid dipeptide (RE) repeats	chr4: 149882952–149883011	84	190	−2.0	0.0013	0.2103

### RT-qPCR validation of regulated genes

**Figure 3:**
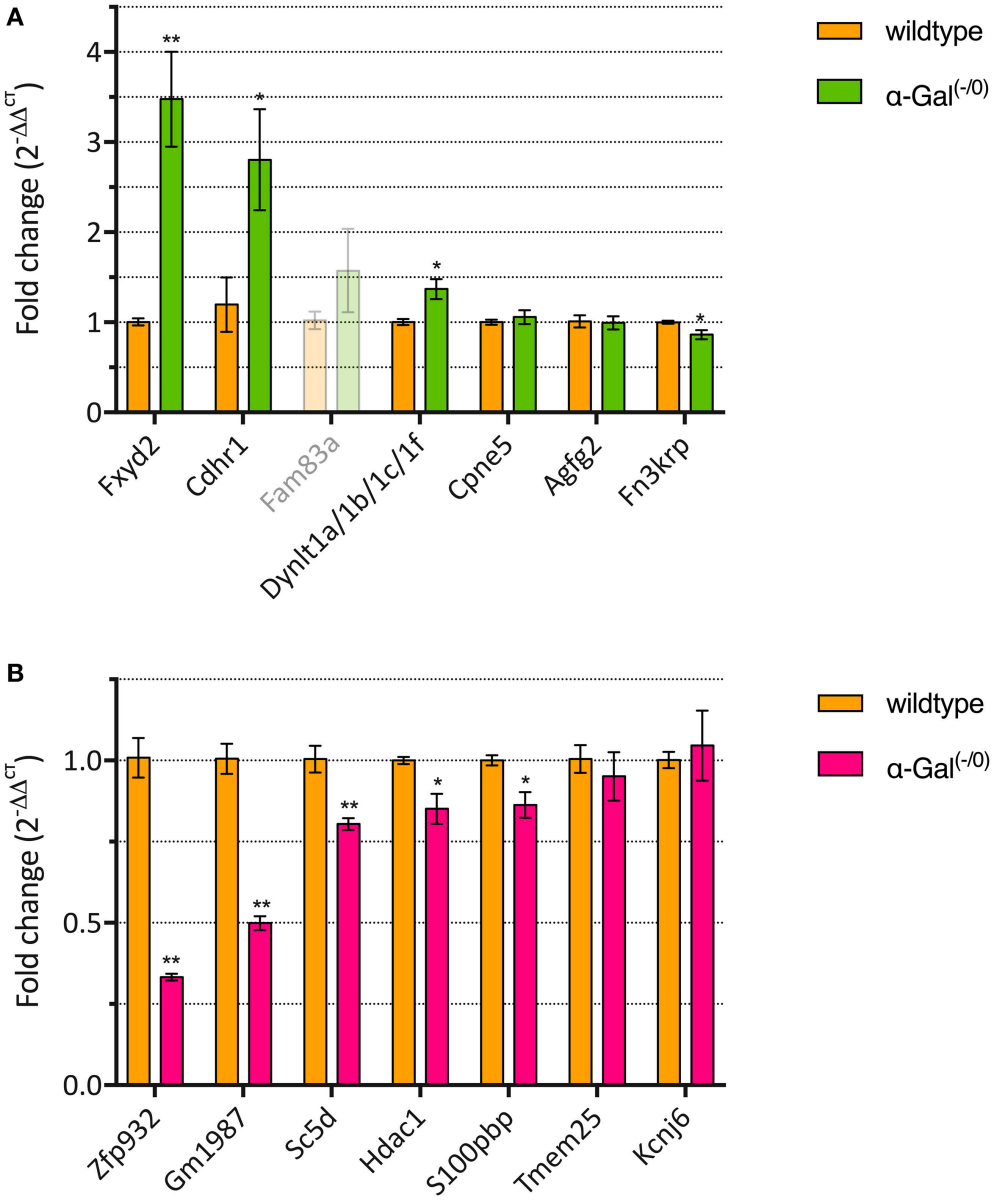
RT-qPCR validation of up- **(A)** and downregulated genes **(B)**. ^*^p < 0.05, ^**^p < 0.01.

We performed RT-qPCR analysis of the top 7 up- and downregulated genes in a separate set of PFC samples from α-Gal A^(−/0)^ mice backcrossed with C57BL/6J mice and control C57BL/6J wildtype mice, to validate the differentially expressed genes from the microarray expression profiling. One of the upregulated genes (i.e., Fam83a) had C_T_-values higher than 35 and was therefore excluded from further analysis. For the remaining upregulated genes, we validated that 3/6 genes (i.e., Fxyd2, Cdhr1, and Dynlt1a/1b/1c/1f) showed significant upregulation (Figure 3A, Supplementary Table 1). Furthermore, 5/7 of the downregulated genes (i.e., Zfp932, Gm1987, Sc5d, Hdac1, and S100pbp) showed significant downregulation (Figure 3B, Supplementary Table 1). Altogether, RT-qPCR validation yielded a verification rate of 62%.

### Enriched pathways and protein-protein interactions for upregulated mRNAs

**Figure 4:**
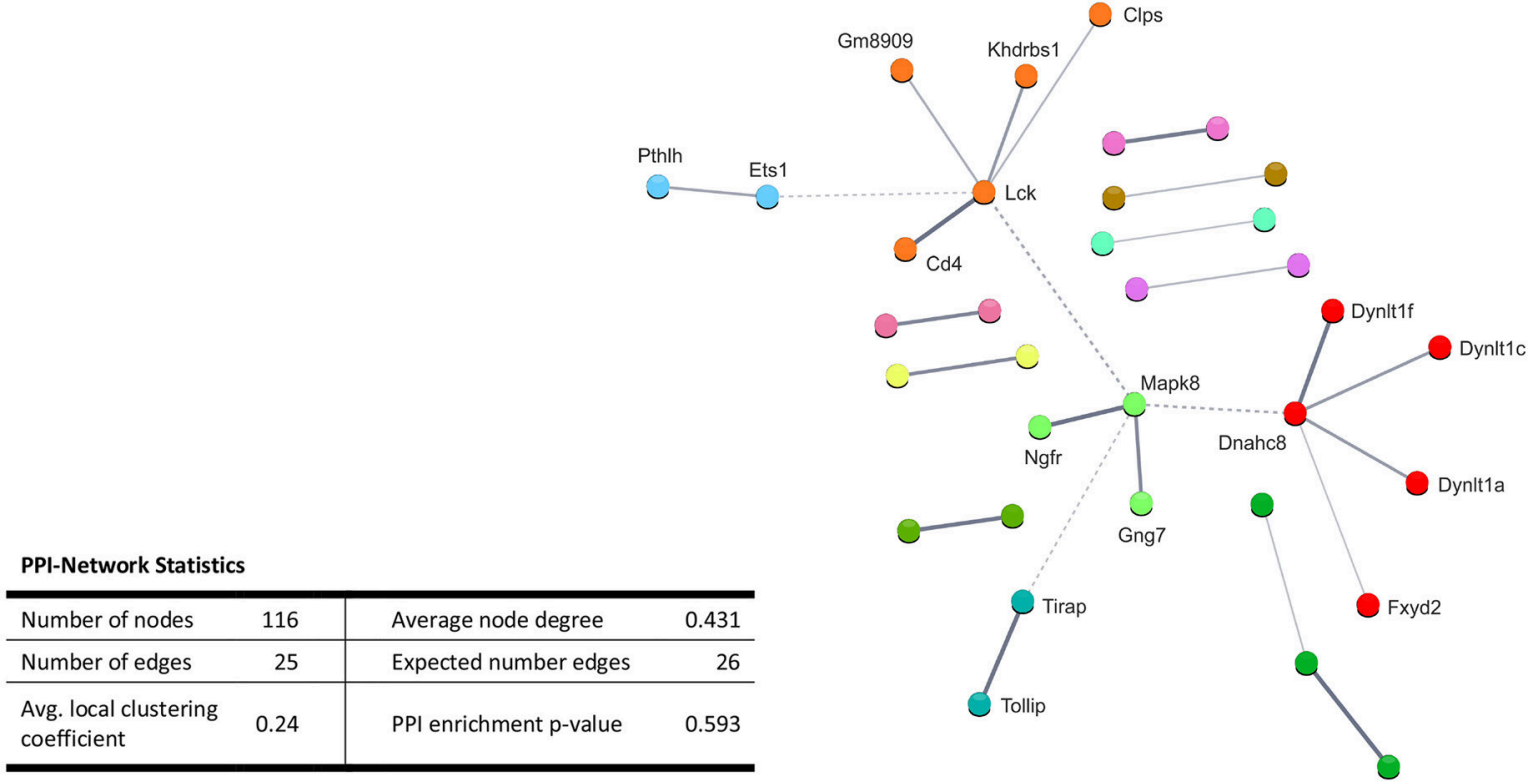
STRING database protein-protein interaction (PPI) networks of significantly *upregulated* genes. Cut-off values, *p* ≤ 0.01, fold change ≥ 1.2. Cluster analysis did not reveal any protein-protein interaction clusters.

Performing enrichment analysis of the 135 upregulated genes neither revealed Gene Ontology processes, nor KEGG pathways. Also, PPI analysis of the significantly upregulated mRNAs revealed no significant PPI enrichment (*p* = 0.59; Figure 4).

### Enriched pathways and protein-protein interactions for downregulated mRNAs

Enrichment analysis of the 246 significantly downregulated genes revealed a number of regulated pathways, including immune related pathways (e.g., immune system process, immune/defense responses, regulation of cytokine production) and signaling pathways (e.g., regulation of cell communication, regulation of signaling, positive regulation of transport; Table 3).

**Table 3:** Enrichment-analysis for *downregulated* mRNAs in α-Gal^(−/0)^ vs. wildtype mice using Gene Ontology and KEGG pathway annotations.

**Pathway ID**	**Pathway description**	**Count in network**	**False discovery rate**
**BIOLOGICAL PROCESSES (GO)**					
GO.0050789	Regulation of biological process	94	0.0382
GO.0050794	Regulation of cellular process	91	0.0355
GO.0048518	Positive regulation of biological process	61	0.0466
GO.0048583	Regulation of response to stimulus	43	0.0350
GO.0051239	Regulation of multicellular organismal process	40	0.0073
GO.0010646	Regulation of cell communication	39	0.0355
GO.0023051	Regulation of signaling	37	0.0455
GO.0002376	Immune system process	35	< 0.0001
GO.0006955	Immune response	26	< 0.0001
GO.0006952	Defense response	22	0.0073
GO.0040011	Locomotion	21	0.0355
GO.0051241	Negative regulation of multicellular organismal process	20	0.0466
GO.0051050	Positive regulation of transport	19	0.0355
GO.0001817	Regulation of cytokine production	17	0.0014
GO.0030155	Regulation of cell adhesion	15	0.0355
GO.0045087	Innate immune response	14	0.0073
GO.0006935	Chemotaxis	13	0.0244
GO.0002252	Immune effector process	12	0.0244
GO.0007159	Leukocyte cell-cell adhesion	10	0.0423
GO.0001818	Negative regulation of cytokine production	9	0.0244
GO.2000249	Regulation of actin cytoskeleton reorganization	4	0.0438
GO.0032606	Type I interferon production	3	0.0355
GO.0002880	Regulation of chronic inflammatory response to non-antigenic stimulus	2	0.0466

*Whole genome was used as statistical background*.

**Figure 5:**
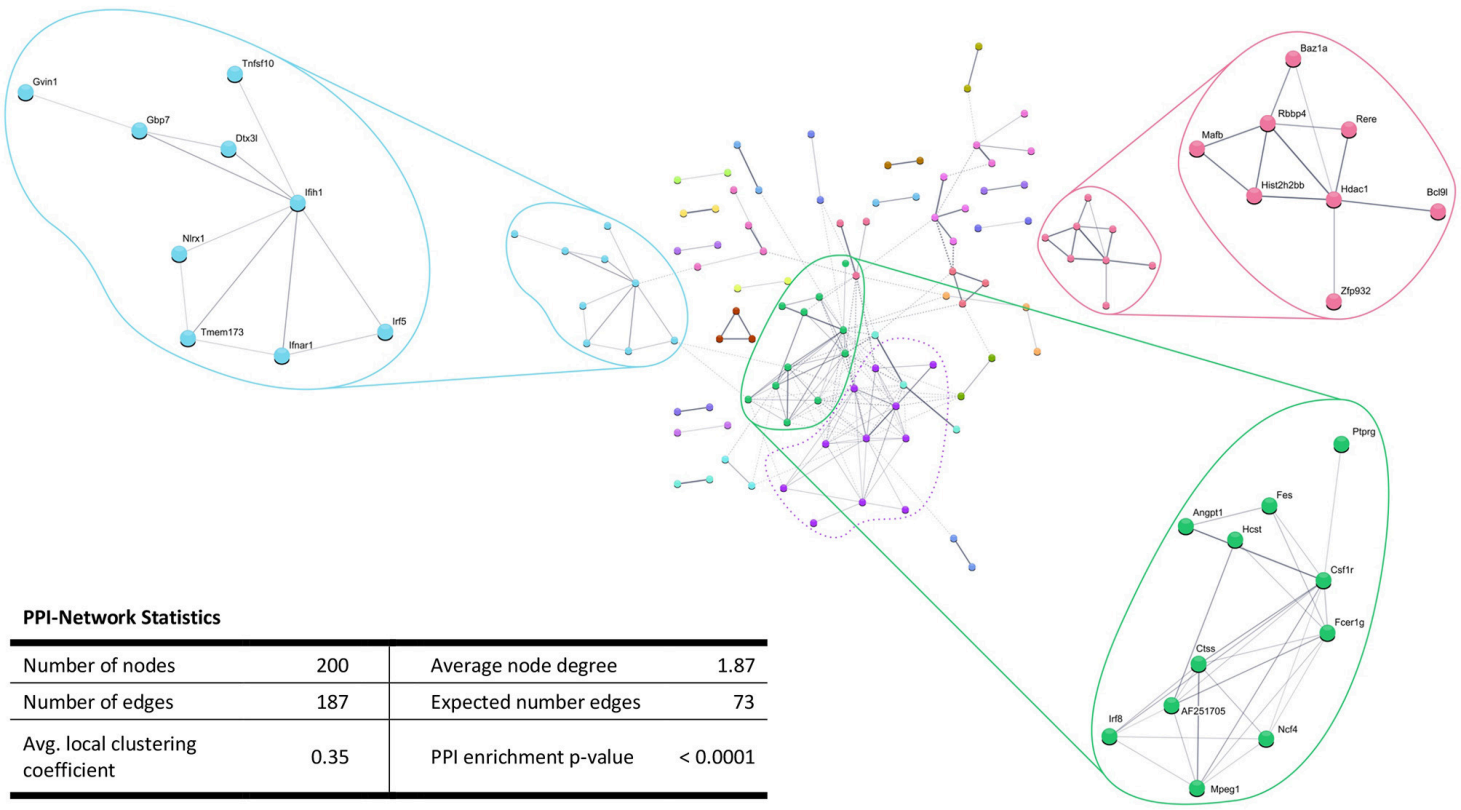
STRING database protein-protein interaction (PPI) networks of significantly *downregulated* genes. Cut-off values, *p* ≤ 0.01, fold change ≥ 1.2. Cluster analysis revealed four clusters of interacting proteins, of which one (purple cluster) did not show significantly enriched pathways and processes.

In addition, the PPI analysis of significantly downregulated mRNAs showed a significant PPI enrichment (*p* < 0.0001; Figure 5). The number of actually observed edges (*n* = 187) exceeded the expected number of edges (*n* = 73) by 156%. When taking a closer look at the PPI network, three clusters of highly interconnected proteins became apparent. Enrichment analysis of these clusters showed that those genes were involved in different pathways (Table 4). The blue and green clusters were related to the immune system (e.g., immune response—GO:0006955, interferon production—GO:0032479, cytokine production—GO:0001817), whereas the pink cluster was associated with DNA modification and gene expression (e.g., gene expression—GO:0010467, DNA binding—GO:0003677).

**Table 4:** GO Biological processes and molecular functions of PPI-clusters from *downregulated* mRNAs in α-Gal^(−/0)^ vs. wildtype mice.

**Pathway ID**	**Pathway description**	**Count in network**	**False discovery rate**
**BLUE CLUSTER**			
GO:0002376	Immune system process	6	0.0054
GO:0006955	Immune response	5	0.0052
GO:0006952	Defense response	5	0.0074
GO:0032479	Regulation of type I interferon production	4	< 0.0001
GO:0051607	Defense response to virus	4	0.0006
GO:0045087	Innate immune response	4	0.0077
GO:0032481	Positive regulation of type I interferon production	3	0.0009
GO:0032648	Regulation of interferon-beta production	3	0.0011
GO:0032606	Type I interferon production	2	0.0054
GO:0032728	Positive regulation of interferon-beta production	2	0.0416
**GREEN CLUSTER**			
GO:0002376	Immune system process	7	0.0069
GO:0032879	Regulation of localization	7	0.0155
GO:0051049	Regulation of transport	6	0.0246
GO:0001817	Regulation of cytokine production	5	0.0069
GO:1903530	Regulation of secretion by cell	5	0.0110
GO:0006955	Immune response	5	0.0155
GO:0051050	Positive regulation of transport	5	0.0239
GO:0001819	Positive regulation of cytokine production	4	0.0155
GO:0006935	Chemotaxis	4	0.0239
GO:0050707	Regulation of cytokine secretion	3	0.0239
**PINK CLUSTER**			
GO:0006351	Transcription, DNA-templated	7	0.0007
GO:0006355	Regulation of transcription, DNA-templated	7	0.0018
GO:0010467	Gene expression	7	0.0027
GO:0003677	DNA binding	6	0.0062
GO:0006357	Regulation of transcription from RNA polymerase II promoter	5	0.0202
GO:0043565	Sequence-specific DNA binding	5	0.0061
GO:0006338	Chromatin remodeling	4	0.0005
GO:0051276	Chromosome organization	4	0.0190
GO:0000976	Transcription Regulatory Region Sequence-Specific DNA binding	4	0.0193
GO:0042826	Histone deacetylase binding	3	0.0061
GO:0000183	Chromatin silencing at rDNA	2	0.0140
GO:0043044	ATP-dependent chromatin remodeling	2	0.0221
GO:0001106	RNA polymerase II transcription corepressor activity	2	0.0223

### Regulation of ion channels, receptors, and signaling proteins

To link the cognitive deficits associated with FD to an impairment of cortical neuron function, we specifically screened the microarray dataset for genes related to ion channels, receptors, synapse and signaling proteins. We found that two potassium channels (i.e., Kcnj13 and Kcnj6), as well as the neuropeptide S (Npsr1) and adenosine A2b receptors (Adora2b) were downregulated (Supplementary Table 2). Validation of some of these differentially expressed genes was, however, problematic possibly due to dilution of neuronally expressed genes by genes from glial or other cell types. The delta chemokine receptor Cx3cr1 was also downregulated. Further, we found that ten different receptor tyrosine kinases (e.g., Il1rap, Il3ra, etc.) and protein tyrosine phosphatases (i.e., Ptprb and Ptprg) were downregulated. Furthermore, a number of synapse and signaling proteins were downregulated in the screen. The mRNA levels of the synaptic proteins Synaptoporin (Synpr), Synaptojanin 2 (Synj2), the active zone protein Rims1 and Snap25, as well as the signaling proteins RAS protein activator Rasal3 and the G-protein signaling modulator Gpsm3 were decreased.

In contrast, the nicotinic acetylcholine receptor α-subunit 4 and the low-density lipoprotein receptor sortillin-related receptor (Sorl1) were upregulated in our screen, as were the toll-interleukin 1 receptor domain-containing adaptor protein (Tirap), the mitogen-activated proteins Mapk8 and Mapk1ip1l, and the G-protein Gng7. These findings suggest that indeed functional changes occur in the brain as a consequence of α-Gal A depletion which, may affect synaptic signaling and information processing in cortical circuits.

## Discussion

FD patients exhibit small fiber neuropathy with pain symptoms already in early childhood, which are thought to arise from accumulation of the glycolipid Gb3 specifically in DRG neurons and peripheral nerves (Kocen and Thomas, 1970; Ohnishi and Dyck, 1974; Bangari et al., 2015; Godel et al., 2017). However, neuronal accumulation of Gb3 not only occurs in DRGs and peripheral nerves, but also in central nervous system tissues, like the spinal cord, medulla oblongata, hypothalamus, amygdala, substantia nigra, raphe nuclei, hippocampus, and cortical layers 5/6 (de Veber et al., 1992; Itoh et al., 2001; Khanna et al., 2010; Tuttolomondo et al., 2013a,b). Besides accumulation in neurons, glycolipid deposits are also found in the walls of cerebral vessels, leading to occlusion of blood vessels with malperfusion of different brain areas (Tagliavini et al., 1982; Itoh et al., 2001; Reisin et al., 2011; Tuttolomondo et al., 2013a), acute ischemia and stroke (Fazekas et al., 2013), microstructural damage and white matter lesions (Fellgiebel et al., 2012; Paavilainen et al., 2013; Sigmundsdottir et al., 2014; Underhill et al., 2015). Even mutations in the GLA gene that were considered asymptomatic are associated with neuronal damage (Lenders et al., 2013).

In line with these findings, cognitive deficits frequently occur in FD patients: Besides decreased health-related quality of life, which itself already constitutes a debilitating factor for affected patients, an increased risk for depression, anxiety, acute psychotic symptoms, as well as personality and behavioral changes is associated with FD (Bolsover et al., 2014). In addition, deficits in general intellectual functioning, speed of information processing, reasoning, verbal fluency and problem solving are frequent (Sigmundsdottir et al., 2014), whereas alterations of memory and attentional performance are controversially discussed: While in a study of Sigmundsdottir et al. (2014) no differences in memory and attention are reported, Schermuly et al. (2011) present evidence for deficits in the attention domain, as well as learning and memory deficits that correlate with the extent of white matter lesions. Also, FD patients can develop progressive and significant hippocampal volume loss over an 8-year observation period (Lelieveld et al., 2015).

Based on these studies on the involvement of circuits responsible for attention and executive function, we for the first time investigated differences in mRNA expression in the PFC of a well-accepted FD mouse model [α-Gal A^(−/0)^]. An FD specific gene expression profile was discovered in murine brain samples with 381 differentially expressed genes. Of those, 135 genes were significantly up-, and 246 were significantly downregulated. Involved pathways comprised mainly immune system processes, cytokine production and cell signaling. This finding correlates well with previous studies in humans, showing that lysosomal storage disorders, and particularly FD, are associated with deficits in innate and adaptive immunity (Daly et al., 2000; Hawkins-Salsbury et al., 2011; Mauhin et al., 2015). Especially the downregulated genes in the green and blue clusters were associated with immune system related pathways (Table 4). Interestingly, age-related cognitive decline is associated with exacerbated neuroinflammatory responses in the aging brain (David et al., 1997; Viviani and Boraso, 2011; Corona et al., 2012; Farso et al., 2013; Xie et al., 2017), and the brains from aged animals show increased cytokine and chemokine levels (Bodles and Barger, 2004; Lynch et al., 2010). These might be caused by activated microglia, which constitute the major source of inflammatory cytokines and chemokines in the central nervous system (Hanisch and Kettenmann, 2007; Kettenmann et al., 2011). So far, however, microglia involvement has not been systematically explored in FD.

Another cluster that emerged in our analysis was associated with DNA binding, chromatin remodeling and gene expression (Table 4, pink cluster). Several studies show that lysosomal storage disorders are associated with epigenetic changes (Hassan et al., 2017). Differential methylation patterns and decreased expression of DNA methyltransferase 3a are found in cerebellar neurons of a mouse model for presymptomatic Niemann-Pick type C disease (Kennedy et al., 2016) and Hubner et al. (2015) provide the only report on alteration of calcitonin receptor promotor methylation in FD patients on enzyme replacement therapy. Histone modifications as well as the efficacy of HDAC inhibitors have been investigated for Niemann-Pick type C (Helquist et al., 2013) and Gaucher disease (Lu et al., 2011). Regarding FD, evidence is available from transgenic mouse fibroblasts overexpressing a human α-Gal A mutation that HDAC inhibition rescues α-Gal A trafficking blockade, but without an effect on lysosomal Gb3 storage (Yam et al., 2007). Finally, another possibility of regulating gene expression is through non-coding RNAs, particularly microRNAs, and dysregulation of microRNAs occurs in the Niemann-Pick type C as well as Gaucher type lysosomal storage disorders (Queiroz et al., 2016). However, respective knowledge on microRNA dysregulation in FD is unavailable to date.

Regarding the cognitive deficits, several ion channels, such as Kcnj13 or Kcnj6, and ion channel modulators were deregulated in the present screen. Some of them, e.g., Kir7.1 (Kcnj13) are critically involved in setting neuronal excitability and firing activity (Ghamari-Langroudi et al., 2015). G protein-coupled inwardly rectifying potassium (GIRK/Kir3) channels such as Girk2 (Kcnj6) are key effectors in inhibitory signaling pathways. GIRK-dependent signaling contributes to pain perception, reward-related behavior, mood, cognition and addiction (Lujan et al., 2014). In particular, several Kcnj6 “pain risk” alleles are known to date (Bruehl et al., 2013), and neuroprotective roles of GIRK channels are emerging (Sanchez-Rodriguez et al., 2017), supporting a potential contribution of Kcnj6 downregulation in the cognitive decline of FD patients with age.

Few of the significantly regulated genes discovered in the present study are associated with aging- or disease-related pathophysiological changes: Neuropeptide S modulates arousal and produces anxiolytic-like effects (Xu et al., 2004; Okamura and Reinscheid, 2007), therefore the downregulation of neuropeptide S receptor (Npsr1) found in the current screen could be linked to the increased anxiety-like behavior found in FD mice (Hofmann et al., 2017). The Interferon alpha/beta receptor Ifnar1 is associated with sickness behavior and cognitive dysfunction, which frequently occurs upon Type 1 Interferon treatment in humans and in patients with autoimmune disorders (Blank et al., 2016), and lack of Ifnar1 causes Lewy Body- and Parkinson's disease-like dementia (Ejlerskov et al., 2015). The histone-binding protein Rbbp4, which was downregulated in our screen, is involved in age-related memory loss, and inhibition of this gene causes hippocampus dependent memory deficits in young mice (Pavlopoulos et al., 2013). Early life stress leads to decreased HDAC1 levels targeting promoters of plasticity-associated genes, e.g., the transcription factor Egr2, while at the same time triggering persistent impairment in working memory and attention (Adler and Schmauss, 2016). Neuronal expression of the protein tyrosine phosphatase Ptprb is involved in neurito- and synaptogenesis (Hayashi et al., 2005), and Interleukin 1 modulates hippocampal neuron function by potentiation of NMDA-induced calcium influx (Huang et al., 2011). The lysosomal cysteine proteinase Ctss and the high affinity IgE receptor gamma subunit Fcer1g, which were both downregulated in the current study, are associated with Alzheimer's disease (AD), and in particular, Fcer1g is considered as a risk factor for AD (Taguchi et al., 2005; Castillo et al., 2017). Ctss is also related to amyotrophic lateral sclerosis (ALS) (Berjaoui et al., 2015). Although ALS represents a neurodegenerative disorder that predominantly affects the motor system, cognitive decline and behavioral symptoms occur in ALS patients (Phukan et al., 2007), and mutations in Macrophage colony-stimulation factor 1 receptor (Csf1r), a gene that interacts with both Ctss and Fcer1g, causes axonal spheroids as a sign of Wallerian degeneration (Lynch et al., 2016). Csf1r, which was downregulated in our screen, facilitates protection and survival of uninjured neurons in the hippocampus and cortex (Luo et al., 2013), and Csf1r signaling via administration of Csf1 ameliorates memory deficits in an Alzheimer's disease mouse model (Boissonneault et al., 2009). Finally, Interleukin 3 has been shown to protect cortical neurons from neurodegeneration (Zambrano et al., 2007). Thus, our findings support the idea that neurodegeneration may constitute a so far neglected entity of FD pathology in the peripheral and the central nervous system.

Interestingly, significantly regulated genes in the current screen were to a large extent similar to those from our recently published gene expression analysis of α-Gal A^(−/0)^ mouse DRGs (Kummer et al., 2017). Similar to DRGs, immune system related pathways (e.g., “immune response”, “defense response”, etc.) were overrepresented. Also, the most differentially expressed genes (i.e., Pmepa1, S100pbp, Tmem25, Hdac1, Zfp932, Pyroxd2, Dynlt1c, Dynlt1f) as well as predicted genes (i.e., Gm1987, Gm3893, Gm13298, 4933409K07Rik, 2310058N22Rik, 1190007I07Rik, A930033H14Rik) were strongly regulated in both PFC and DRG samples. Altogether, 113 individual genes were significantly regulated in both neuronal tissue types, suggesting a pan-neuronal effect that might be a hallmark and the underlying cause of neuron abnormalities leading to changes in excitability and signal transduction in FD.

Particularly strenuous for FD patients are the pain attacks they experience already in adolescence (Germain, 2010). In addition to the altered gene expression in DRGs, gene expression changes in relevant brain areas may play a critically important part in the development of the FD pain phenotype. Both prefrontal and sensory cortices, but also insular cortex and basal ganglia circuits are actively involved in the perception of chronic neuropathic pain (Xie et al., 2009), and white matter lesions affecting projections to these brain regions are likely involved in the amplified pain perception of FD patients. Irrespective of whether the cognitive deficits in FD are directly caused by a neuronal dysfunction due to changes in gene expression and regulation, or if they are caused indirectly by deficits in blood supply of specific brain regions, they constitute an important clinical problem.

Different limitations have to be considered when evaluating the current data analysis. Although the α-Gal A^(−/0)^ mouse model closely resembles the underlying genetics of FD patients, alternative FD mouse models have been developed, as for example the G3Stg/GLA^(−/−)^ mouse, which expresses human Gb3 synthase (Taguchi et al., 2013), or the NOD/SCID immune deficiency mouse that also shows tissue specific Gb3 accumulation, although without clinical manifestation (Pacienza et al., 2012). It would be important to investigate if similar genes and pathways are affected in those mouse models. Also, the general moderate statistical power of gene expression screens due to the low number of tested cases—in the current experiment four per group—should be considered. Although the genotype of inbred genetically modified mouse lines can be assumed to be similar, genetic variations between individuals can exist. When performing gene expression screens in small cohorts, the probability of false positive results is high, due to the large number of multiple comparisons performed for all genes tested. Therefore, different methods of FDR (false discovery rate) corrections have been introduced for large scale genetic studies. These FDR corrections drastically reduce the number of false positively reported changed genes, but on the other hand dramatically increase the number of false negatives (Park and Mori, 2010). As a result, genes that are actually differentially expressed might be judged as not being significantly changed, and therefore might be disregarded in subsequent validation experiments. Exceedingly stringent correction of gene expression analysis over-emphasizes true positive genes and shifts the results toward a true-positive/false-negative bias. The presented analysis was designed to nevertheless provide a weighed analysis, neither overestimating false-positives, nor false-negatives, due to the moderate stringency applied.

In the present screen we make use of a genetic FD mouse model to provide first knowledge on gene expression signatures and pathways other than Gb3 accumulation in central nervous system neuronal tissue that may be involved in FD pathogenesis.

## Author contributions

KK, ML and MK designed the study. KK, TK, ML and MM performed the data collection, analyzed and interpreted the data. KK and MK wrote the manuscript. TK, MM and ML critically reviewed the contents of the paper and suggested substantial improvements. All authors have approved the final version of the manuscript.

### Conflict of interest statement

The authors declare that the research was conducted in the absence of any commercial or financial relationships that could be construed as a potential conflict of interest.

## References

[B1] Adler S. M. Schmauss C. (2016). Cognitive deficits triggered by early life stress: the role of histone deacetylase 1. Neurobiol. Dis. 94, 1–9. 10.1016/j.nbd.2016.05.018 27260837 PMC4983517

[B2] Bangari D. S. Ashe K. M. Desnick R. J. Maloney C. Lydon J. Piepenhagen P.. (2015). Alpha-galactosidase a knockout mice: progressive organ pathology resembles the type 2 later-onset phenotype of Fabry disease. Am. J. Pathol. 185, 651–665. 10.1016/j.ajpath.2014.11.004 25553976

[B3] Benjamini Y. Hochberg Y. (1995). Controlling the false discovery rate - a practical and powerful approach to multiple testing. J. R. Stat. Soc. Ser. B Methodol. 57, 289–300.

[B4] Berjaoui S. Povedano M. Garcia-Esparcia P. Carmona M. Aso E. Ferrer I. (2015). Complex inflammation mRNA-related response in ALS is region dependent. Neural Plast. 2015:573784 10.1155/2015/573784 26301107 PMC4537753

[B5] Blank T. Detje C. N. Spiess A. Hagemeyer N. Brendecke S. M. Wolfart J.. (2016). Brain endothelial- and epithelial-specific interferon receptor chain 1 drives virus-induced sickness behavior and cognitive impairment. Immunity 44, 901–912. 10.1016/j.immuni.2016.04.005 27096319

[B6] Bodles A. M. Barger S. W. (2004). Cytokines and the aging brain - what we don't know might help us. Trends Neurosci. 27, 621–626. 10.1016/j.tins.2004.07.011 15374674

[B7] Boissonneault V. Filali M. Lessard M. Relton J. Wong G. Rivest S. (2009). Powerful beneficial effects of macrophage colony-stimulating factor on beta-amyloid deposition and cognitive impairment in Alzheimer's disease. Brain 132, 1078–1092. 10.1093/brain/awn331 19151372

[B8] Bolsover F. E. Murphy E. Cipolotti L. Werring D. J. Lachmann R. H. (2014). Cognitive dysfunction and depression in Fabry disease: a systematic review. J. Inherit. Metab. Dis. 37, 177–187. 10.1007/s10545-013-9643-x 23949010

[B9] Bruehl S. Denton J. S. Lonergan D. Koran M. E. Chont M. Sobey C.. (2013). Associations between KCNJ6 (GIRK2) gene polymorphisms and pain-related phenotypes. Pain 154, 2853–2859. 10.1016/j.pain.2013.08.026 23994450 PMC3845348

[B10] Castillo E. Leon J. Mazzei G. Abolhassani N. Haruyama N. Saito T.. (2017). Comparative profiling of cortical gene expression in Alzheimer's disease patients and mouse models demonstrates a link between amyloidosis and neuroinflammation. Sci. Rep. 7:17762. 10.1038/s41598-017-17999-3 29259249 PMC5736730

[B11] Cocozza S. Pontillo G. Quarantelli M. Sacca F. Riccio E. Costabile T.. (2018). Default mode network modifications in Fabry disease: a resting-state fMRI study with structural correlations. Hum. Brain Mapp. 39, 1755–1764. 10.1002/hbm.23949 29315984 PMC6866450

[B12] Corona A. W. Fenn A. M. Godbout J. P. (2012). Cognitive and behavioral consequences of impaired immunoregulation in aging. J. Neuroimmune Pharmacol. 7, 7–23. 10.1007/s11481-011-9313-4 21932047

[B13] Daly T. M. Lorenz R. G. Sands M. S. (2000). Abnormal immune function *in vivo* in a murine model of lysosomal storage disease. Pediatr. Res. 47, 757–762. 10.1203/00006450-200006000-00012 10832733

[B14] David J. P. Ghozali F. Fallet-Bianco C. Wattez A. Delaine S. Boniface B.. (1997). Glial reaction in the hippocampal formation is highly correlated with aging in human brain. Neurosci. Lett. 235, 53–56. 10.1016/S0304-3940(97)00708-8 9389594

[B15] Desnick R. J. Ioannou Y. A. Eng C. M. (2001). alpha-galactosidase a deficiency: fabry disease, in The Metabolic and Molecular Bases of Inherited Disease, eds Scriver C. R. Beaudet A. L. Sly W. S. Valle D. (New York, NY: McGraw-Hill), 3733–3774.

[B16] de Veber G. A. Schwarting G. A. Kolodny E. H. Kowall N. W. (1992). Fabry disease: immunocytochemical characterization of neuronal involvement. Ann. Neurol. 31, 409–415. 10.1002/ana.410310410 1375013

[B17] Edgar R. Domrachev M. Lash A. E. (2002). Gene expression omnibus: NCBI gene expression and hybridization array data repository. Nucleic Acids Res. 30, 207–210. 10.1093/nar/30.1.207 11752295 PMC99122

[B18] Ejlerskov P. Hultberg J. G. Wang J. Carlsson R. Ambjorn M. Kuss M.. (2015). Lack of neuronal IFN-beta-IFNAR causes lewy body- and parkinson's disease-like dementia. Cell 163, 324–339. 10.1016/j.cell.2015.08.069 26451483 PMC4601085

[B19] Farso M. Menard C. Colby-Milley J. Quirion R. (2013). Immune marker CD68 correlates with cognitive impairment in normally aged rats. Neurobiol. Aging 34, 1971–1976. 10.1016/j.neurobiolaging.2013.02.008 23523271

[B20] Fazekas F. Enzinger C. Schmidt R. Dichgans M. Gaertner B. Jungehulsing G. J.. (2013). MRI in acute cerebral ischemia of the young: the Stroke in Young Fabry Patients (sifap1) Study. Neurology 81, 1914–1921. 10.1212/01.wnl.0000436611.28210.ec 24186912

[B21] Fellgiebel A. Wolf D. O. Kolodny E. Muller M. J. (2012). Hippocampal atrophy as a surrogate of neuronal involvement in Fabry disease. J. Inherit. Metab. Dis. 35, 363–367. 10.1007/s10545-011-9390-9 21932096

[B22] Gal A. Schafer E. Rohard I. (2006). The genetic basis of Fabry disease, in Fabry Disease: Perspectives from 5 Years of FOS, eds Mehta A. Beck M. Sunder-Plassmann G. (Oxford: Oxford PharmaGenesis)

[B23] Germain D. P. (2010). Fabry disease. Orphanet J. Rare Dis. 5:30 10.1186/1750-1172-5-30 21092187 PMC3009617

[B24] Ghamari-Langroudi M. Digby G. J. Sebag J. A. Millhauser G. L. Palomino R. Matthews R.. (2015). G-protein-independent coupling of MC4R to Kir7.1 in hypothalamic neurons. Nature 520, 94–98. 10.1038/nature14051 25600267 PMC4383680

[B25] Godel T. Baumer P. Pham M. Kohn A. Muschol N. Kronlage M.. (2017). Human dorsal root ganglion *in vivo* morphometry and perfusion in Fabry painful neuropathy. Neurology 89, 1274–1282. 10.1212/WNL.0000000000004396 28835396

[B26] Hanisch U. K. Kettenmann H. (2007). Microglia: active sensor and versatile effector cells in the normal and pathologic brain. Nat. Neurosci. 10, 1387–1394. 10.1038/nn1997 17965659

[B27] Hassan S. Sidransky E. Tayebi N. (2017). The role of epigenetics in lysosomal storage disorders: uncharted territory. Mol. Genet. Metab. 122, 10–18. 10.1016/j.ymgme.2017.07.012 28918065

[B28] Hawkins-Salsbury J. A. Reddy A. S. Sands M. S. (2011). Combination therapies for lysosomal storage disease: is the whole greater than the sum of its parts? Hum. Mol. Genet. 20, R54–R60. 10.1093/hmg/ddr112 21421999 PMC3095053

[B29] Hayashi N. Miyata S. Yamada M. Kamei K. Oohira A. (2005). Neuronal expression of the chondroitin sulfate proteoglycans receptor-type protein-tyrosine phosphatase β and phosphacan. Neuroscience 131, 331–348. 10.1016/j.neuroscience.2004.11.017 15708477

[B30] Helquist P. Maxfield F. R. Wiech N. L. Wiest O. (2013). Treatment of niemann–pick type C disease by histone deacetylase inhibitors. Neurotherapeutics 10, 688–697. 10.1007/s13311-013-0217-2 24048860 PMC3805865

[B31] Hofmann L. Karl F. Sommer C. Uceyler N. (2017). Affective and cognitive behavior in the alpha-galactosidase a deficient mouse model of Fabry disease. PLoS ONE 12:e0180601. 10.1371/journal.pone.0180601 28662189 PMC5491260

[B32] Huang Y. Smith D. E. Ibanez-Sandoval O. Sims J. E. Friedman W. J. (2011). Neuron-specific effects of interleukin-1β are mediated by a novel isoform of the IL-1 receptor accessory protein. J. Neurosci. 31, 18048–18059. 10.1523/JNEUROSCI.4067-11.2011 22159118 PMC3261076

[B33] Hubner A. Metz T. Schanzer A. Greber-Platzer S. Item C. B. (2015). Aberrant DNA methylation of calcitonin receptor in Fabry patients treated with enzyme replacement therapy. Mol. Genet. Metab. Rep. 5, 1–2. 10.1016/j.ymgmr.2015.08.002 28649534 PMC5471449

[B34] Itoh Y. Esaki T. Cook M. Qasba P. Shimoji K. Alroy J.. (2001). Local and global cerebral blood flow and glucose utilization in the alpha-galactosidase a knockout mouse model of Fabry disease. J. Neurochem. 79, 1217–1224. 10.1046/j.1471-4159.2001.00669.x 11752062

[B35] Kennedy B. E. Hundert A. S. Goguen D. Weaver I. C. Karten B. (2016). Presymptomatic alterations in amino acid metabolism and DNA methylation in the cerebellum of a murine model of niemann-pick type c disease. Am. J. Pathol. 186, 1582–1597. 10.1016/j.ajpath.2016.02.012 27083515

[B36] Kettenmann H. Hanisch U. K. Noda M. Verkhratsky A. (2011). Physiology of microglia. Physiol. Rev. 91, 461–553. 10.1152/physrev.00011.2010 21527731

[B37] Khanna R. Soska R. Lun Y. Feng J. Frascella M. Young B.. (2010). The pharmacological chaperone 1-deoxygalactonojirimycin reduces tissue globotriaosylceramide levels in a mouse model of Fabry disease. Mol. Ther. 18, 23–33. 10.1038/mt.2009.220 19773742 PMC2839206

[B38] Kocen R. S. Thomas P. K. (1970). Peripheral nerve involvement in fabrys disease. Arch. Neurol. 22, 81–88. 10.1001/archneur.1970.00480190085014 4311670

[B39] Kono Y. Wakabayashi T. Kobayashi M. Ohashi T. Eto Y. Ida H.. (2016). Characteristics of cerebral microbleeds in patients with fabry disease. J. Stroke Cerebrovasc. Dis. 25, 1320–1325. 10.1016/j.jstrokecerebrovasdis.2016.02.019 26987491

[B40] Kummer K. K. Kalpachidou T. Kress M. Langeslag M. (2017). Signatures of altered gene expression in dorsal root ganglia of a fabry disease mouse model. Front. Mol. Neurosci. 10:449 10.3389/fnmol.2017.00449 29422837 PMC5788883

[B41] Lelieveld I. M. Bottcher A. Hennermann J. B. Beck M. Fellgiebel A. (2015). Eight-year follow-up of neuropsychiatric symptoms and brain structural changes in fabry disease. PLoS ONE 10:e0137603. 10.1371/journal.pone.0137603 26340726 PMC4560446

[B42] Lenders M. Duning T. Schelleckes M. Schmitz B. Stander S. Rolfs A.. (2013). Multifocal white matter lesions associated with the D313Y mutation of the α-galactosidase a gene. PLoS ONE 8:e55565. 10.1371/journal.pone.0055565 23393592 PMC3564750

[B43] Lu J. Yang C. Chen M. Ye D. Y. Lonser R. R. Brady R. O.. (2011). Histone deacetylase inhibitors prevent the degradation and restore the activity of glucocerebrosidase in Gaucher disease. Proc. Natl. Acad. Sci. U.S.A. 108, 21200–21205. 10.1073/pnas.1119181109 22160715 PMC3248545

[B44] Lujan R. Marron Fernandez De Velasco E. Aguado C. Wickman K. (2014). New insights into the therapeutic potential of Girk channels. Trends Neurosci. 37, 20–29. 10.1016/j.tins.2013.10.006 24268819 PMC3880623

[B45] Luo J. Elwood F. Britschgi M. Villeda S. Zhang H. Ding Z.. (2013). Colony-stimulating factor 1 receptor (CSF1R) signaling in injured neurons facilitates protection and survival. J. Exp. Med. 210, 157–172. 10.1084/jem.20120412 23296467 PMC3549715

[B46] Lynch A. M. Murphy K. J. Deighan B. F. O'reilly J. A. Gun'ko Y. K. Cowley T. R.. (2010). The impact of glial activation in the aging brain. Aging Dis. 1, 262–278. 22396865 PMC3295033

[B47] Lynch D. S. Zhang W. J. Lakshmanan R. Kinsella J. A. Uzun G. A. Karbay M.. (2016). Analysis of mutations in AARS2 in a series of CSF1R-negative patients with adult-onset leukoencephalopathy with axonal spheroids and pigmented glia. JAMA Neurol. 73, 1433–1439. 10.1001/jamaneurol.2016.2229 27749956

[B48] Mauhin W. Lidove O. Masat E. Mingozzi F. Mariampillai K. Ziza J. M.. (2015). Innate and adaptive immune response in fabry disease. JIMD Rep. 22, 1–10. 10.1007/8904_2014_371 25690728 PMC4486269

[B49] Mechtler T. P. Stary S. Metz T. F. De Jesus V. R. Greber-Platzer S. Pollak A.. (2012). Neonatal screening for lysosomal storage disorders: feasibility and incidence from a nationwide study in Austria. Lancet 379, 335–341. 10.1016/S0140-6736(11)61266-X 22133539

[B50] Ohnishi A. Dyck P. J. (1974). Loss of small peripheral sensory neurons in fabry disease - histologic and morphometric evaluation of cutaneous nerves, spinal ganglia, and posterior columns. Arch. Neurol. 31, 120–127. 10.1001/archneur.1974.00490380068009 4135101

[B51] Ohshima T. Murray G. J. Swaim W. D. Longenecker G. Quirk J. M. Cardarelli C. O.. (1997). Alpha-galactosidase a deficient mice: a model of Fabry disease. Proc. Natl. Acad. Sci. U.S.A. 94, 2540–2544. 10.1073/pnas.94.6.2540 9122231 PMC20124

[B52] Okamura N. Reinscheid R. K. (2007). Neuropeptide S: a novel modulator of stress and arousal. Stress 10, 221–226. 10.1080/10253890701248673 17613937

[B53] Paavilainen T. Lepomaki V. Saunavaara J. Borra R. Nuutila P. Kantola I.. (2013). Diffusion tensor imaging and brain volumetry in Fabry disease patients. Neuroradiology 55, 551–558. 10.1007/s00234-012-1131-8 23292181

[B54] Pacienza N. Yoshimitsu M. Mizue N. Au B. C. Wang J. C. Fan X.. (2012). Lentivector transduction improves outcomes over transplantation of human HSCs alone in NOD/SCID/Fabry mice. Mol. Ther. 20, 1454–1461. 10.1038/mt.2012.64 22472949 PMC3393855

[B55] Park B. S. Mori M. (2010). Balancing false discovery and false negative rates in selection of differentially expressed genes in microarrays. Open Access Bioinformatics 2010, 1–9. 10.2147/OAB.S7181 22022206 PMC3197253

[B56] Pavlopoulos E. Jones S. Kosmidis S. Close M. Kim C. Kovalerchik O.. (2013). Molecular mechanism for age-related memory loss: the histone-binding protein RbAp48. Sci. Transl. Med. 5:200ra115. 10.1126/scitranslmed.3006373 23986399 PMC4940031

[B57] Phukan J. Pender N. P. Hardiman O. (2007). Cognitive impairment in amyotrophic lateral sclerosis. Lancet Neurol. 6, 994–1003. 10.1016/S1474-4422(07)70265-X 17945153

[B58] Queiroz M. T. Pereira V. G. Do Nascimento C. C. D'almeida V. (2016). The underexploited role of non-coding RNAs in lysosomal storage diseases. Front. Endocrinol. 7:133. 10.3389/fendo.2016.00133 27708618 PMC5030823

[B59] R Development Core Team (2008). R: A Language and Environment for Statistical Computing. Vienna: R Foundation for Statistical Computing.

[B60] Reisin R. C. Romero C. Marchesoni C. Napoli G. Kisinovsky I. Caceres G.. (2011). Brain MRI findings in patients with Fabry disease. J. Neurol. Sci. 305, 41–44. 10.1016/j.jns.2011.03.020 21463870

[B61] Saito S. Ohno K. Sakuraba H. (2011). Fabry-database.org: database of the clinical phenotypes, genotypes and mutant alpha-galactosidase a structures in Fabry disease. J. Hum. Genet. 56, 467–468. 10.1038/jhg.2011.31 21412250

[B62] Sanchez-Rodriguez I. Temprano-Carazo S. Najera A. Djebari S. Yajeya J. Gruart A.. (2017). Activation of G-protein-gated inwardly rectifying potassium (Kir3/GirK) channels rescues hippocampal functions in a mouse model of early amyloid-beta pathology. Sci. Rep. 7:14658. 10.1038/s41598-017-15306-8 29116174 PMC5676742

[B63] Schermuly I. Muller M. J. Muller K. M. Albrecht J. Keller I. Yakushev I.. (2011). Neuropsychiatric symptoms and brain structural alterations in Fabry disease. Eur. J. Neurol. 18, 347–353. 10.1111/j.1468-1331.2010.03155.x 20636371

[B64] Sigmundsdottir L. Tchan M. C. Knopman A. A. Menzies G. C. Batchelor J. Sillence D. O. (2014). Cognitive and psychological functioning in Fabry disease. Arch. Clin. Neuropsychol. 29, 642–650. 10.1093/arclin/acu047 25319043 PMC4263929

[B65] Spada M. Pagliardini S. Yasuda M. Tukel T. Thiagarajan G. Sakuraba H.. (2006). High incidence of later-onset fabry disease revealed by newborn screening. Am. J. Hum. Genet. 79, 31–40. 10.1086/504601 16773563 PMC1474133

[B66] Szklarczyk D. Morris J. H. Cook H. Kuhn M. Wyder S. Simonovic M.. (2017). The STRING database in 2017: quality-controlled protein-protein association networks, made broadly accessible. Nucleic Acids Res. 45, D362–D368. 10.1093/nar/gkw937 27924014 PMC5210637

[B67] Tagliavini F. Pietrini V. Gemignani F. Lechi A. Pallini R. Federico A. (1982). Anderson-Fabry's disease: neuropathological and neurochemical investigation. Acta Neuropathol. 56, 93–98. 10.1007/BF00690579 6278815

[B68] Taguchi A. Maruyama H. Nameta M. Yamamoto T. Matsuda J. Kulkarni A. B.. (2013). A symptomatic Fabry disease mouse model generated by inducing globotriaosylceramide synthesis. Biochem. J. 456, 373–383. 10.1042/BJ20130825 24094090 PMC4160053

[B69] Taguchi K. Yamagata H. D. Zhong W. Kamino K. Akatsu H. Hata R.. (2005). Identification of hippocampus-related candidate genes for Alzheimer's disease. Ann. Neurol. 57, 585–588. 10.1002/ana.20433 15786443

[B70] Tuttolomondo A. Pecoraro R. Simonetta I. Miceli S. Arnao V. Licata G.. (2013a). Neurological complications of Anderson-Fabry disease. Curr. Pharm. Des. 19, 6014–6030. 10.2174/13816128113199990387 23448452

[B71] Tuttolomondo A. Pecoraro R. Simonetta I. Miceli S. Pinto A. Licata G. (2013b). Anderson-Fabry disease: a multiorgan disease. Curr. Pharm. Des. 19, 5974–5996. 10.2174/13816128113199990352 23448451

[B72] Underhill H. R. Golden-Grant K. Garrett L. T. Uhrich S. Zielinski B. A. Scott C. R. (2015). Detecting the effects of Fabry disease in the adult human brain with diffusion tensor imaging and fast bound-pool fraction imaging. J. Magn. Reson. Imaging 42, 1611–1622. 10.1002/jmri.24952 26018987 PMC4662657

[B73] Viviani B. Boraso M. (2011). Cytokines and neuronal channels: a molecular basis for age-related decline of neuronal function? Exp. Gerontol. 46, 199–206. 10.1016/j.exger.2010.09.008 20869430

[B74] Wilcox W. R. Oliveira J. P. Hopkin R. J. Ortiz A. Banikazemi M. Feldt-Rasmussen U.. (2008). Females with Fabry disease frequently have major organ involvement: lessons from the Fabry Registry. Mol. Genet. Metab. 93, 112–128. 10.1016/j.ymgme.2007.09.013 18037317

[B75] Xie Y. F. Huo F. Q. Tang J. S. (2009). Cerebral cortex modulation of pain. Acta Pharmacol. Sin. 30, 31–41. 10.1038/aps.2008.14 19079295 PMC4006538

[B76] Xie Z. M. Wang X. M. Xu N. Wang J. Pan W. Tang X. H.. (2017). Alterations in the inflammatory cytokines and brain-derived neurotrophic factor contribute to depression-like phenotype after spared nerve injury: improvement by ketamine. Sci. Rep. 7:3124. 10.1038/s41598-017-03590-3 28600519 PMC5466642

[B77] Xu Y. L. Reinscheid R. K. Huitron-Resendiz S. Clark S. D. Wang Z. Lin S. H.. (2004). Neuropeptide S: a neuropeptide promoting arousal and anxiolytic-like effects. Neuron 43, 487–497. 10.1016/j.neuron.2004.08.005 15312648

[B78] Yam G. H. Roth J. Zuber C. (2007). 4-Phenylbutyrate rescues trafficking incompetent mutant α-galactosidase A without restoring its functionality. Biochem. Biophys. Res. Commun. 360, 375–380. 10.1016/j.bbrc.2007.06.048 17592721

[B79] Zambrano A. Otth C. Mujica L. Concha I.i Maccioni R. B. (2007). Interleukin-3 prevents neuronal death induced by amyloid peptide. BMC Neurosci. 8:82. 10.1186/1471-2202-8-82 17915029 PMC2089076

