# Supplementary material for: Altered Gene Expression in Prefrontal Cortex of a Fabry Disease Mouse Model
Source: Front Mol Neurosci. 2018 Jun 25;11:201. doi: 10.3389/fnmol.2018.00201 (PMC6036252; doi:10.3389/fnmol.2018.00201)
Supplement: Supplementary file 1 [file Data_Sheet_1.docx]

**Supplementary Table 1.** RT-qPCR validation of up- and downregulated genes.

|  | *Mean ± SEM* | | *Welch-corrected t-test* | | |
| --- | --- | --- | --- | --- | --- |
| *Gene* | *wildtype* | *α-Gal^(-/0)^* | *t* | *df* | *p-value* |
| Fxyd2 | 1.004 ± 0.0390 | 3.477 ± 0.5273 | 4.68 | 5.06 | 0.0053 |
| Cdhr1 | 1.197 ± 0.3015 | 2.805 ± 0.5596 | 2.53 | 7.68 | 0.0364 |
| Fam83a | 1.022 ± 0.0982 | 1.574 ± 0.4636 | 1.17 | 4.36 | 0.3038 |
| Dynlt1a/1b/1c/1f | 1.002 ± 0.0342 | 1.370 ± 0.1107 | 3.17 | 5.93 | 0.0196 |
| Cpne5 | 1.002 ± 0.0278 | 1.058 ± 0.0764 | 0.69 | 6.30 | 0.5142 |
| Agfg2 | 1.012 ± 0.0672 | 0.994 ± 0.0721 | 0.18 | 9.95 | 0.8641 |
| Fn3krp | 1.001 ± 0.0164 | 0.863 ± 0.0517 | 2.55 | 6.00 | 0.0437 |
|  |  |  |  |  |  |
| Zfp932 | 1.008 ± 0.0606 | 0.333 ± 0.0101 | 11.01 | 5.28 | < 0.0001 |
| Gm1987 | 1.006 ± 0.0463 | 0.499 ± 0.0213 | 9.94 | 6.95 | < 0.0001 |
| Sc5d | 1.004 ± 0.0415 | 0.804 ± 0.0179 | 4.43 | 6.79 | 0.0033 |
| Hdac1 | 1.000 ± 0.0108 | 0.852 ± 0.0464 | 3.12 | 5.54 | 0.0228 |
| S100pbp | 1.001 ± 0.0160 | 0.863 ± 0.0393 | 3.24 | 6.62 | 0.0154 |
| Tmem25 | 1.005 ± 0.0423 | 0.951 ± 0.0746 | 0.62 | 7.92 | 0.5502 |
| Kcnj6 | 1.002 ± 0.0250 | 1.046 ± 0.1078 | 0.40 | 5.54 | 0.7038 |
|  |  |  |  |  |  |

**Supplementary Table 2.** Raw expression values, fold changes and statistical analysis for significantly regulated ion channels, receptors and signaling proteins.

|  |  |  | |  | |  | |  | |  | |  | |  |
| --- | --- | --- | --- | --- | --- | --- | --- | --- | --- | --- | --- | --- | --- | --- |
| *NCBI RefSeq ID* | *Gene Symbol* | *Gene Name* | | *Expression α-Gal^-/0^* | | *Expression wildtype* | | *fold change* | | *p-value* | | *FDR* | |  |
|  |  |  | |  | |  | |  | |  | |  | |  |
| ***Ion channels and receptors*** | |  | |  | |  | |  | |  | |  | |  |
| NM_011436 | Sorl1 | sortilin-related receptor, LDLR class A repeats-containing | | 14319 | | 9508 | | 1.8 | | 0.0004 | | 0.1160 | |  |
| NM_015730.5 | Chrna4 | cholinergic receptor, nicotinic, alpha polypeptide 4 | | 1119 | | 929 | | 1.4 | | 0.0062 | | 0.3613 | |  |
| NM_001110227 | Kcnj13 | potassium inwardly-rectifying channel, subfamily J, member 13 | | 368 | | 498 | | -1.2 | | 0.0035 | | 0.2946 | |  |
| NM_009987 | Cx3cr1 | chemokine (C-X3-C) receptor 1 | | 4692 | | 6704 | | -1.2 | | 0.0057 | | 0.3473 | |  |
| NM_007413 | Adora2b | adenosine A2b receptor | | 407 | | 588 | | -1.3 | | 0.0031 | | 0.2892 | |  |
| AK162665 | Sorl1 | sortilin-related receptor, LDLR class A repeats-containing | | 5086 | | 7610 | | -1.3 | | 0.0023 | | 0.2604 | |  |
| NM_175678 | Npsr1 | neuropeptide S receptor 1 | | 61 | | 128 | | -1.9 | | 0.0000 | | 0.0289 | |  |
| NM_001025585 | Kcnj6 | potassium inwardly-rectifying channel, subfamily J, member 6 | | 91 | | 322 | | -3.2 | | 0.0000 | | 0.0050 | |  |
|  |  |  | |  | |  | |  | |  | |  | |  |
| ***Receptor tyrosine kinases and protein tyrosine phosphatases*** | | | | |  | |  | |  | |  | |  | |
| NM_054096 | Tirap | | toll-interleukin 1 receptor (TIR) domain-containing adaptor protein | | 202 | | 140 | | 1.6 | | 0.0072 | | 0.3839 | |
| NM_029928 | Ptprb | | protein tyrosine phosphatase, receptor type, B | | 3470 | | 5067 | | -1.3 | | 0.0005 | | 0.1266 | |
| NM_029928 | Ptprb | | protein tyrosine phosphatase, receptor type, B | | 242 | | 356 | | -1.3 | | 0.0076 | | 0.3873 | |
| NM_001037859 | Csf1r | | colony stimulating factor 1 receptor | | 913 | | 1340 | | -1.3 | | 0.0011 | | 0.1961 | |
| NM_001037859 | Csf1r | | colony stimulating factor 1 receptor | | 15248 | | 24103 | | -1.4 | | 0.0014 | | 0.2149 | |
| AK083209 | Il1rap | | interleukin 1 receptor accessory protein | | 623 | | 958 | | -1.4 | | 0.0013 | | 0.2125 | |
| NM_008981 | Ptprg | | protein tyrosine phosphatase, receptor type, G | | 796 | | 1227 | | -1.4 | | 0.0097 | | 0.4169 | |
| NM_010185 | Fcer1g | | Fc receptor, IgE, high affinity I, gamma polypeptide | | 1401 | | 2197 | | -1.4 | | 0.0096 | | 0.4159 | |
| NM_008369 | Il3ra | | interleukin 3 receptor, alpha chain | | 490 | | 963 | | -1.7 | | 0.0000 | | 0.0289 | |
| NM_001077189 | Fcgr2b | | Fc receptor, IgG, low affinity IIb | | 82 | | 159 | | -1.7 | | 0.0017 | | 0.2337 | |
| NM_010508 | Ifnar1 | | interferon (alpha and beta) receptor 1 | | 1764 | | 3605 | | -1.8 | | 0.0000 | | 0.0298 | |
|  |  | |  | |  | |  | |  | |  | |  | |
| ***Synapse and signaling proteins*** | | |  | |  | |  | |  | |  | |  | |
| NM_016700 | Mapk8 | | mitogen-activated protein kinase 8 | | 970 | | 791 | | 1.4 | | 0.0002 | | 0.0711 | |
| NM_178684 | Mapk1ip1l | | mitogen-activated protein kinase 1 interacting protein 1-like | | 284 | | 237 | | 1.3 | | 0.0022 | | 0.2522 | |
| NM_001038655 | Gng7 | | guanine nucleotide binding protein (G protein), gamma 7 | | 277 | | 235 | | 1.3 | | 0.0039 | | 0.3067 | |
| NM_011428 | Snap25 | | synaptosomal-associated protein 25 | | 49393 | | 69996 | | -1.2 | | 0.0088 | | 0.4071 | |
| NM_134116 | Gpsm3 | | G-protein signalling modulator 3 (AGS3-like, C. elegans) | | 628 | | 894 | | -1.3 | | 0.0089 | | 0.4074 | |
| NM_053270 | Rims1 | | regulating synaptic membrane exocytosis 1 | | 438 | | 638 | | -1.3 | | 0.0024 | | 0.2604 | |
| NM_011523 | Synj2 | | synaptojanin 2 | | 445 | | 716 | | -1.4 | | 0.0068 | | 0.3745 | |
| NM_001163032 | Synpr | | synaptoporin | | 172 | | 274 | | -1.4 | | 0.0069 | | 0.3785 | |
| NM_028638 | Gadl1 | | glutamate decarboxylase-like 1 | | 52 | | 101 | | -1.7 | | 0.0029 | | 0.2832 | |
| NM_178785 | Rasal3 | | RAS protein activator like 3 | | 72 | | 144 | | -1.8 | | 0.0056 | | 0.3466 | |
|  |  | |  | |  | |  | |  | |  | |  | |
